# Hybrid_DBP: Prediction of DNA-binding proteins using hybrid features and convolutional neural networks

**DOI:** 10.3389/fphar.2022.1031759

**Published:** 2022-10-10

**Authors:** Shaoyou Yu, Dejun Peng, Wen Zhu, Bo Liao, Peng Wang, Dongxuan Yang, Fangxiang Wu

**Affiliations:** ^1^ Key Laboratory of Computational Science and Application of Hainan Province, Haikou, China; ^2^ Key Laboratory of Data Science and Intelligence Education, Hainan Normal University, Ministry of Education, Haikou, China; ^3^ School of Mathematics and Statistics, Hainan Normal University, Haikou, China

**Keywords:** DNA-binding proteins, monoDiKGap, CC-PSSM, kmer, MRMD2.0, convolutional neural network

## Abstract

DNA-binding proteins (DBP) play an essential role in the genetics and evolution of organisms. A particular DNA sequence could provide underlying therapeutic benefits for hereditary diseases and cancers. Studying these proteins can timely and effectively understand their mechanistic analysis and play a particular function in disease prevention and treatment. The limitation of identifying DNA-binding protein members from the sequence database is time-consuming, costly, and ineffective. Therefore, efficient methods for improving DBP classification are crucial to disease research. In this paper, we developed a novel predictor Hybrid _DBP, which identified potential DBP by using hybrid features and convolutional neural networks. The method combines two feature selection methods, MonoDiKGap and Kmer, and then used MRMD2.0 to remove redundant features. According to the results, 94% of DBP were correctly recognized, and the accuracy of the independent test set reached 91.2%. This means Hybrid_ DBP can become a useful prediction tool for predicting DBP.

## 1 Introduction

DNA-related activities are integral to biological cellular life activities, including detecting DNA damage, DNA replication, and gene transcription. The replication and recombination of DNA are facilitated by DNA-binding proteins (Luscombe et al., 2000). The proteins associated with and regulating the life activities of DNA are called DNA-binding proteins (DBP) (Gao and Jeffrey, 2008). In the past few years, DBP has become the subject of increasing research that is crucial to genetics and evolution. Thus, identifying DNA sequences could potentially treat cancer and hereditary diseases. DBP has been determined using several experimental approaches (comprising filter binding analysis, genetic analysis, and X-ray) (Nakano et al., 2016). This technique can provide detailed information about DBP, but it is costly and takes longer to perform. In this post-genomic era, there are many protein sequences containing DNA-binding domains, so how to identify these proteins efficiently and effectively is an important topic worth studying in depth in bioinformatics. The identification and prediction methods of DBP were mostly based on machine learning, and many studies tried to use protein sequence features and machine learning to distinguish DBP. DBD-Threader (Mu and Skolnick, 2009) thread-based approach was applied to predict DNA-binding domains and associated functional sites. DBPBIND (Szilágyi and Skolnick, 2006) identified DBP from amino acid sequences and low-resolution junctions. A support vector machine (SVM) model was constructed by DNABinder (Kumar et al., 2007) by analyzing amino acids and dipeptides. DNA-Prot (Kumar et al., 2009) was originally trained to identify DBP from features derived from sequences using a random forest (RF) classifier, and iDNA-Prot (Lin et al., 2011) was subsequently named. IDNAPro-PseAAC (Liu et al., 2015a) used the SVM to improve the predictive power. After conducting dimensionality reduction, the model was renamed iDNA-Prot|dis (Liu et al., 2014). Kmer1 + ACC (Liu et al., 2016) proposed a new approach combining support vector machines and self-crossing covariance transformations. In DBPred (Lou et al., 2014), the selection was performed by using a mixture of Random Forest and Gaussian Naive Bayes. PsePSSM+PSSM-AB+PSSM-DWT (Lu et al., 2020) was constructed using evolutionary computation and the SVM approach. Protein identification work is increasingly being carried out with artificial intelligence and big data, which brings new perspectives. Many studies have attempted to use deep learning algorithms to distinguish the sequences of identifying DBP (Zhang et al., 2020; Li et al., 2021). Previous approaches have contributed to the development of this field, but the prediction accuracy has not yet reached satisfactory results and needs to be further improved. We urgently need a method for extracting and classifying optimal features to identify DBP.

**FIGURE 1:**
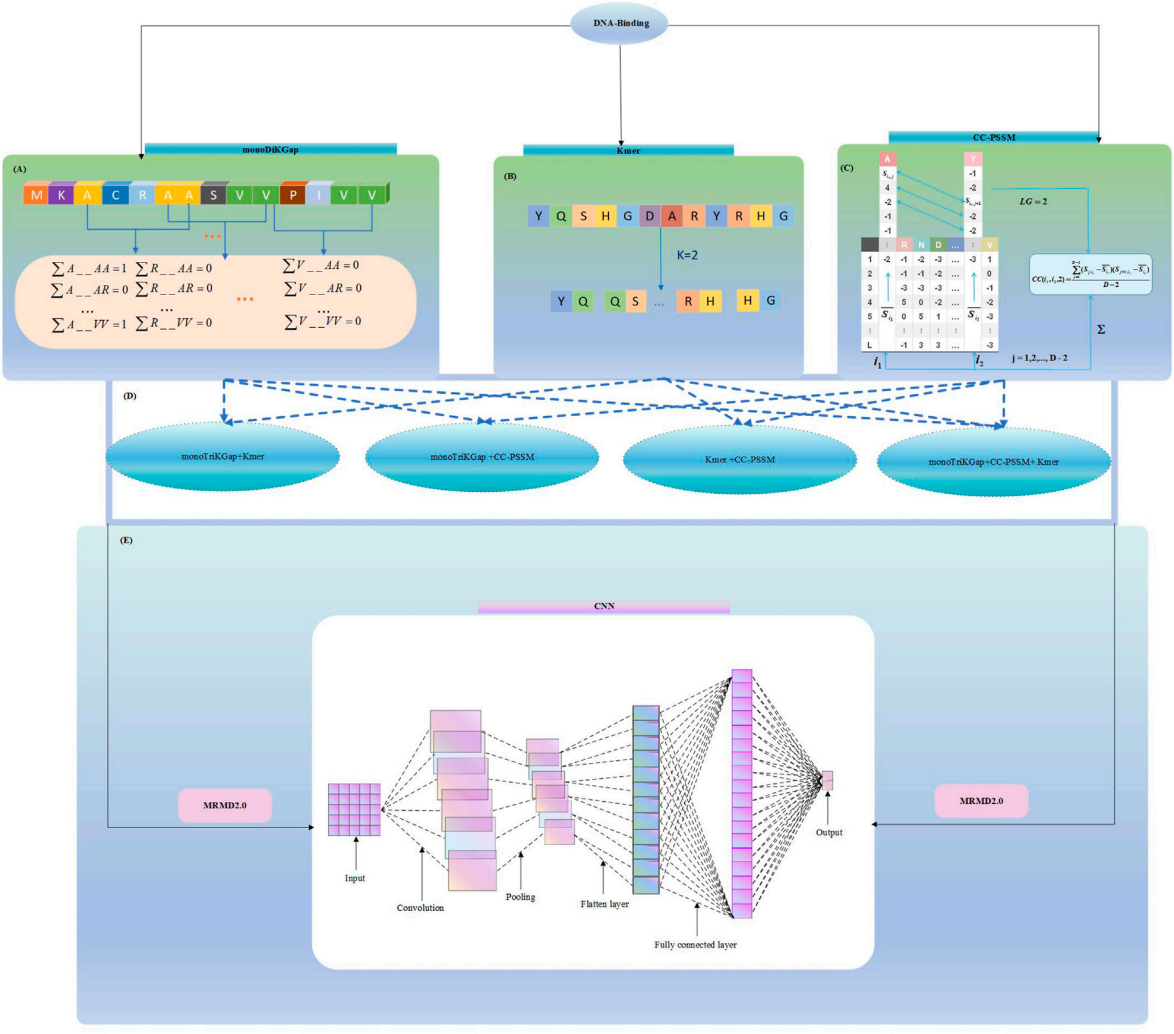
Hybrid_DBP model structure flowchart **(A)** Extract features using monoDiKGap feature selection **(B)** Extract features using Kmer feature selection **(C)** Extract features using CC-PSSM feature selection **(D)** Combine three single feature representations and choose the best hybrid features **(E)** Remove redundant features using MRMD2.0 and Predict DBP by 1-D CNN model.

In this paper, three feature selection approaches were chosen: monoDiKGap, profile-based cross covariance (CC-PSSM) and Kmer. Combining the three features in different combinations was conducted using a hybrid feature approach. We then used MRMD2.0 to remove redundant hybrid features, and then use the convolutional neural networks (CNN) to predict DBP. The result showed the CNN can be very effective in predicting DBP on the basis of the hybrid features of monoDiKGap and Kmer methods. Further, a test set was applied to assess the generalization capability, and the results demonstrated that the model was robust and generalizable. It showed the reliability of this paper’s method for studying DNA-binding proteins. We illustrate our framework in Figure 1, which explains our modeling process.

## 2 Materials and methods

### 2.1 Dataset construction

Datasets of high standard are the foundation for a reliable model. The dataset used in this paper was obtained from Liu et al, (2014), which was collected through the Protein Data Bank (PDB) database of DBP. The database is the most prevalent in the field of bioinformatics. To process the data set further, we deleted the sequences comprising nonstandard amino acid characters “B,” “J,” “O,” “U,” “X,” and “Z.” Finally, 1069 DNA-binding protein samples were obtained, of which 525 were DBP and 544 were non-DBP. To further test the reliability of the model, this paper used Lou et al, (2014) compiling the DNA-binding protein dataset PDB186 as an independent test set, which includes 93 DBPs and 93 non-DBPs. The model data can be downloaded from https://github.com/YUshunL/DBP-file.

### 2.2 Feature selection

#### 2.2.1 MonoDiKGap

The monoDiKGap feature selection is a modification of the kmer feature selection approach in the PyFeat (Rafsanjani et al., 2019). Kmer, a typical and important approach for extracting local features is known as k-tuples. KGap describes a sequence with monoDiKGap combined with subsequences. MonoDiKGap then uses AdaBoost (Zhu et al., 2006) to eliminate redundant features to produce optimal features. The AdaBoost uses the SCRIT package in Python in order to select the n highest scoring features for training after the data have been selected.

As a result of the optimal set of features generated, the dimensionality of the features will be reduced and good predictions will be made. KGap was set at 2 in this study. In monoDiKGap, we can express it as follows:

$$V_{KGap}={\left[f_{1}^{k_{1}},f_{2}^{k_{1}},\ldots ,f_{8000}^{k_{1}},f_{1}^{k_{2}},f_{2}^{k_{2}},\ldots ,f_{8000}^{k_{2}}\right]}^{T}.$$ (1)

In Eq. 1, $f_{i}^{k_{1}}\left(i=1,2,...,8000\right)$ denotes ith feature’s frequency computed, when KGap=1 . $f_{i}^{k_{2}}\left(i=1,2,...,8000\right)$ denotes ith feature’s frequency computed, when KGap=2 . In this way, AdaBoost automatically optimized the total feature set created by 16,000 features and ultimately generated 441 subsets of features.

#### 2.2.2 Profile–based cross covariance (CC-PSSM)

CC-PSSM (Yanzhi et al., 2008) uses a site-specific scoring matrix as a feature. Using PSI-BLAST (Altschul et al., 1997) and NCBI’s NR database, DNA-binding protein sequences were compared with local information to determine PSSM matrix information. Using the PSSM matrix, protein sequences can be predicted based on evolution. The component $S_{ji}$ in the Eq. 2 PSSM matrix indicates the replacement score of the amino acid i in the sequence j.

The CC-PSSM algorithm converts PSSM matrixes of various sizes into vectors of identical length. A difference in property between two residues was computed using CC, along with a lag in sequence between them. Following is the formula:

$$CC\left(i_{1},i_{2},LG\right)=\overset{L-LG}{\underset{j=1}{\sum }}\left(S_{j,i_{1}}-\bar{S_{i_{1}}}\right)\left(S_{j+LG,i_{2}}-\bar{S_{i_{2}}}\right)/\left(L-LG\right).$$ (2)

In the Eq. 2, i1,i2 describes two various amino acids, ${\bar{S}}_{i1},{\bar{S}}_{i2}$ describes the mean of replacement scores for i1,i2 . L is the protein sequence length, and the maximum LG (LG=1,2…,lag) value is lag. In our study, lag is set at 2. Consequently, the protein samples were transformed into 760-length vectors by using computational methods.

#### 2.2.3 Kmer

The Kmer (Liu et al., 2015b) method is a method of extracting protein features based on sequence data, a relatively simple and widely used feature extraction method in bioinformatics. Kmer is a vector consisting of K adjacent amino acid frequency values. $A_{i}$ indicates the amino acid at the i position and $A_{i}\in \left\{A,C,D,E,F,...,W,Y\right\}$ contains the known 20 amino acids. Eqs. 3–4 list the specific features of the two commonly used Kmer-K models (Liu et al., 2017).

$$Kmer-1=\left\{A_{1},A_{2},A_{3},A_{4},...,A_{L-1},A_{L}\right\},$$ (3)

$$Kmer-2=\{A_{1}A_{1},A_{1}A_{2},A_{1}A_{3},...,A_{L}A_{L}\}.$$ (4)

Separate frequencies are calculated for each amino acid arrangement, and the size of the generated frequency vector is $20^{K}$ (Galili, 2015). In this paper, we use K=2 to obtain a 400-dimensional feature vector.

### 2.3 Feature selection

Hundreds of features are selected by feature extraction methods. Nevertheless, some of these features are redundant. In this section, we used the maximum correlation maximum distance (MRMD2.0) (He et al., 2020) to perform feature selection. MRMD2.0 reduces dimensionality and ranks features by identifying those contributing most to predictor variables or outputs. After extracting features from the sequences, it used the concepts of the PageRank algorithm and combined the method coefficients of ANOVA, minimum redundancy and maximum correlation, maximum information, and the minimum absolute shrinkage and selection operators (Quan et al., 2016). Thus, MRMD2.0 used a forward addition method to detect optimized dimensions, combining seven various feature ranking methods with PageRank. Each target page was assigned a weight value according to the PageRank method. Smaller weight values were displayed at the back of pages with larger weight values.

### 2.4 Classification algorithm

This study is a representative binary classification issue to predict DBP. We mainly used CNN algorithms to better explore the prediction model. To better demonstrate the robustness of our model, the three most prominent deep learning (DL) architectures and five classical machine learning (ML) models were compared. In our study, the DL architectures included CNN, Recurrent Neural Network (RNN) (Arunkumar et al., 2021), Long Short- Term Memory (LSTM) (Liu et al., 2021), while the classical ML models included RF (Qi, 2012), SVM (Meng et al., 2020), Naive Bayes (NB) (Sun, 2005), Logistic Regression (LR) (Hosmer et al., 1997)and K-nearest neighbors (KNN) (Samanthula et al., 2015).

A classical CNN has four kinds of layers: convolutional, pooling, flat, and fully connected. A feature extraction process is conducted on the first two layers, while the last two layers map the extracted features to the final output shown in its classical structure (Le et al., 2018; Nguyen et al., 2019). According to the current study, CNN used the following layers:

1) As part of the convolution process, convolution layers were used to extract features embedded in 1D (one dimension) input vectors. Every input shape was transformed with a sliding window and a specific step shift. A representative value was generated by sliding the input shapes. During the convolution process, a vector preserved distances between values. By utilizing small slides of the input data, this layer was learned the important features (Le et al., 2017; Le et al., 2019).
2) The activation layer was performed after the convolution layer. Rectified Linear Unit (ReLU) is a non-linear operation applied and computed in Eq. 5:

f(x)=max(0,x). (5)

In the Eq. 5, x is the amount of inputs. By introducing ReLU into our CNN, we enabled it to learn more effectively based on data analysis.

3) The pooling layer was used for the convolutional layer to decrease the computation of the following layer. Our architecture selected the maximum pooling from three different types of pooling layers to select the maximum value over 2 windows.
4) To cope with the overfitting problem of neural networks and improve the model’s generalization ability, we set the dropout size to 0.2, randomly discarded some neurons, and improved the performance results in some cases.
5) In the flat layer, the previous layer’s feature matrix is flattened into one-dimensional feature vectors, which facilitate the input to the fully connected layer, which is typically found at the end of a CNN network, and consists of individual nodes connected to the inputs (Srivastava et al., 2014).
6) The fully connected layer was typically applied at the end of the neural network. There is a full connection between each node in this layer and every other node during the previous layer. Two layers were fully connected in the current model. Using this first one, we gained more knowledge and enhanced our model’s performance by connecting all input nodes to the spreading layer. The second layer connected this layer to the output layer. Since DNA-binding proteins were classified using binary classification, the output layer consists of 2 nodes.
7) Softmax is an evaluation function that determines the probability of each possible output at the output. The following formula can be used to calculate its function:

$$\sigma {\left(z\right)}_{i}=\frac{e^{z_{i}}}{\sum_{k=1}^{K}e^{z_{k}}}.$$ (6)

A K-dimensional vector represents the input vector z, the ith class is based on predicted probability for x, and $\sigma {\left(z\right)}_{i}$ is real values in the range (0, 1).

### 2.5 Performance evaluation

In this paper, accuracy (ACC) (Wang et al., 2019), sensitivity (SN), specificity (SP) (Zhu et al., 2020), and Mathew correlation coefficient (MCC) (Zeng et al., 2019) were applied as a measure of model performance and represented in Eq. 7–9:

$$SN=\frac{TP}{TP+FN},$$ (7)

$$\text{SP}=\frac{\text{TN}}{\text{TN}+\text{FP}},$$ (8)

$$\text{ACC}=\frac{\text{TP}+\text{TN}}{\text{TP}+\text{TN}+\text{FP}+\text{FN}},$$ (9)

$$\text{MCC}=\frac{\text{TN}\times \text{TP}-\text{FP}\times \text{FN}}{\sqrt{\left(\text{TP}+\text{FN}\right)\left(\text{TP}+\text{FP}\right)\left(\text{TN}+\text{FP}\right)\left(\text{TN}+\text{FN}\right)}},$$ (10)

In the model, TP indicates that the DBP is accurately predicted; FN indicates that the DBP is inaccurately predicted as the non-DBP; FP demonstrates that the model inaccurately predicts the DBP from the non-DBP, and TN demonstrates that the model accurately predicts the non-DBP. To determine the performance of our model, we calculated the area under the ROC curve (AUC) and the precision-recall curve (PRC).

## 3 Results and discussion

### 3.1 Comparative analysis of different characterization methods

**FIGURE 2:**
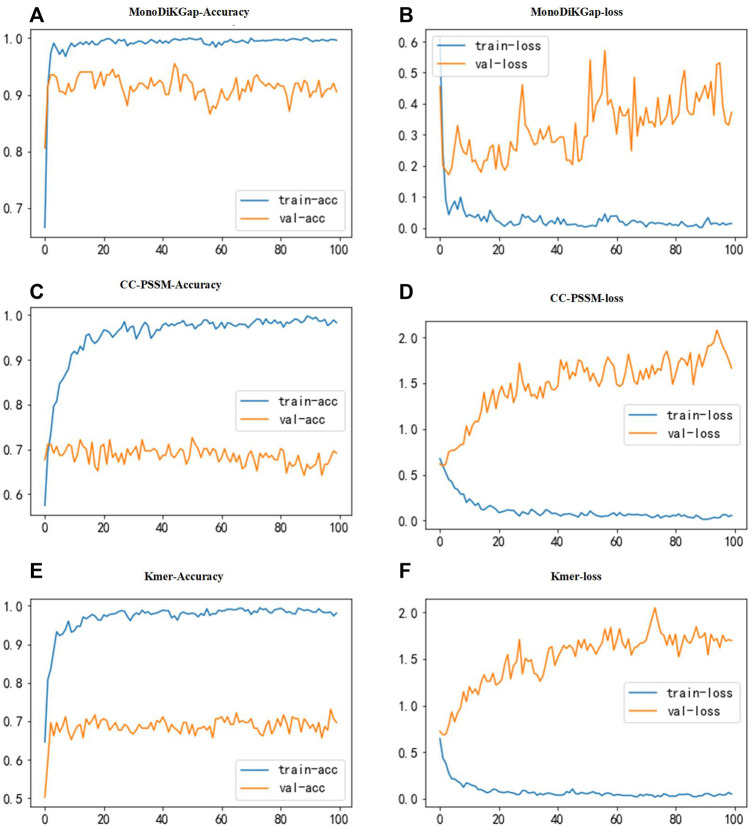
Accuracy and loss function plots for three feature selection methods. **(A,B)** illustrate the accuracy and loss function plots under monoTriKGap. **(C,D)** illustrate the accuracy and loss function plots under CC-PSSM. **(E,F)** illustrate the classification accuracy and loss function plots under Kmer. The blue line describes the training set and the yellow line describes the validation set.

Three feature selection approaches were used for comparison to find better feature types. To illustrate the stability and robustness, we used 80% of the samples in the dataset for training and 20% for validation. The constructed neural network has three convolutional layers. The first has a size in convolutional kernels is 10 and a number of 256, the second has a size in convolutional kernels is 5 and a number of 128, and the third has a size in convolutional kernels is 5 and 64. The convolutional layer’s activation function used ReLU to improve generalization to deal with the neural network’s overfitting problem. The size of the dropout was 0.2, and some neurons were randomly discarded. For the pooling layer, max-pooling was chosen for our model. Figure 2 shows the results of classification using a 1D CNN under the three feature selection methods. We found that the model built based on the 441 optimal feature subsets selected by monoDiKGap has a higher performance than other methods. MonoDiKGap feature extraction method predicted 90.5% for the validation set, which is significantly better than CC-PSSM and Kmer-2. According to the analysis, monoTriKGap outperformed better than other two feature selection methods for classification with 1D CNN. The results illustrated the importance of choosing an appropriate feature extraction method for model building.

### 3.2 Performance comparison of different optimizers methods

**FIGURE 3:**
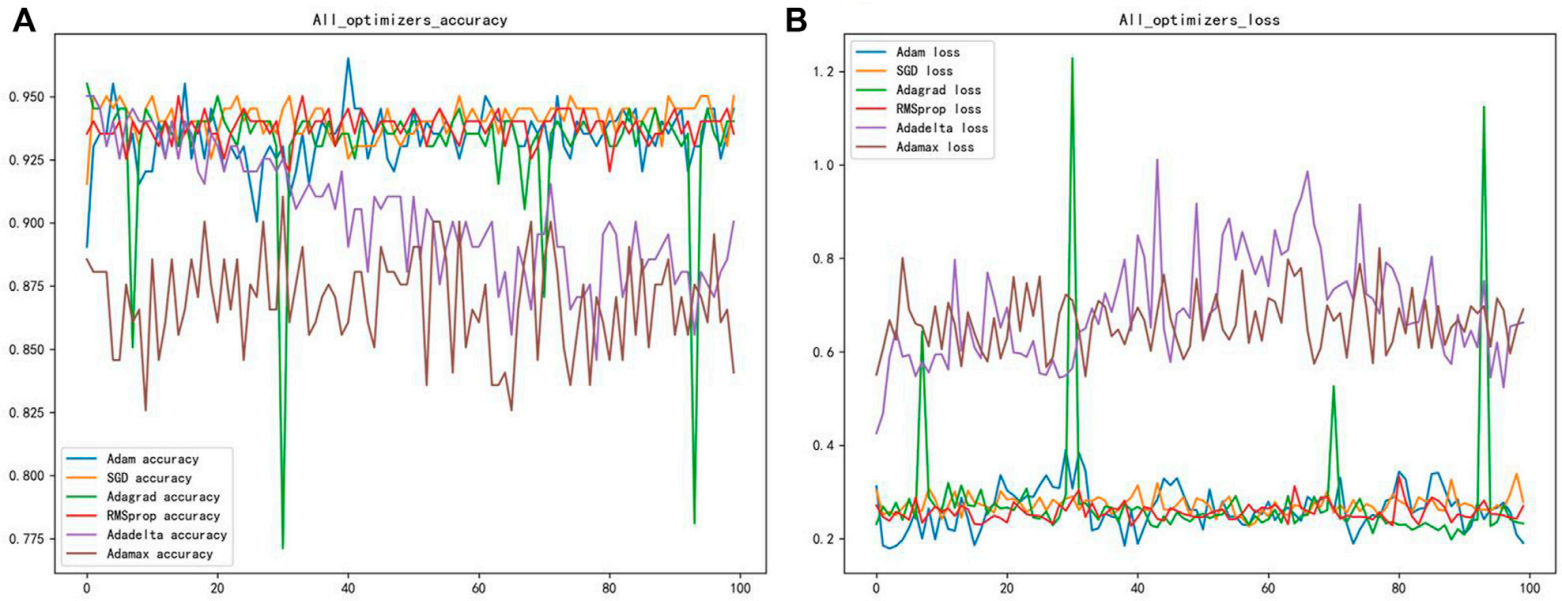
**(A)** Shows the classification accuracy of different optimizers **(B)** shows the loss function of different optimizers.

Different gradient optimization algorithms were applied to optimize the neural network to enhance the accuracy and convergence of the model. Six optimizers were selected in this paper to optimize the weight coefficients and bias coefficients of the neural network, namely: Adam, SGD, Adagrad, RMSprop, Adadelta, and Adamax optimizers (Chensi et al., 2018). The purpose is to find the appropriate optimizer to converge the model faster and better. The accuracy and loss function plots of the six optimizers are shown after 50 epochs in Figure 3. The best optimizer sought should result in the fastest convergence of the model’s accuracy and loss function. Figure 3 showed that the Adam optimizer worked better, with fewer fluctuations, and was more stable than the other optimizers. Therefore, this parameter optimization step is vital for improving the overall model’s accuracy.

### 3.3 Comparison of hybrid feature representation methods

To determine a better method for extracting features for DBP, multiple types of feature information was combined by feature combination. Combining the three feature selection approaches yields four types of feature combination methods: monoTriKGap+CC-PSSM, K-mer2 +CC-PSSM, CC-PSSM + Kmer-2, monoTriKGap+CC-PSSM+K-mer2. There may be redundancies among these feature combinations, which can affect performance. Therefore, each of these four feature combinations was dimensionally reduced through MRMD2.0 and the classification results of the validation set were evaluated using a 1-D CNN, and the results were shown in Table 1. The best classification was found by comparing experimental results when monoTriKGap and Kmer were applied with MRMD2.0 dimensionality reduction, which provided ACC values of 94%, MCC values of 0.91, SN values of 95%, and SP values of 93.1%, which was a 3.5% increase over the accuracy obtained by monoTriKGap with a single optimal feature. The monoDiKGap method combines basic features from adjacent amino acids, generating features to capture amino acid arrangement frequency distributions in biological protein sequences, as well as reducing the amount of dimensionality, complexity and computation time by selecting features with the most distinguishable information based on AdaBoost. An advantage of a K-mer protein representation is that it does not require structural knowledge in order to determine the frequency of incidence of k neighboring amino acids. Howerver, with frequency-based features, the more features that are redundant, the less improvement it makes, and the longer the feature vector becomes, the less generalization ability of the underlying prediction model will be achieved. The CC-PSSM has the advantages of storing the evolutionary information of protein sequences. However, calculating features takes time and does not utilize evolutionary information and protein sequence order information, making the process more time consuming. To analyze the amino acid frequency distribution from different perspectives, we found that combining K-mer and monoDiKGap is more effective, and we called this model Hybrid_DBP.

**TABLE 1:** Performance comparison of different feature combinations under 1-D CNN

**Methods**	**ACC(%)**	**MCC**	**SN(%)**	**SP(%)**
MonoDiKGap+Kmer	94.0	0.909	95.0	93.1
MonoDiKGap+CC-PSSM	78.6	0.572	79.0	78.2
Kmer + CC-PSSM	69.7	0.393	68.0	71.3
MonoDiKGap+Kmer+CC-PSSM	86.1	0.720	86.0	86.1

### 3.4 Stability and generalization capability of models

**FIGURE 4:**
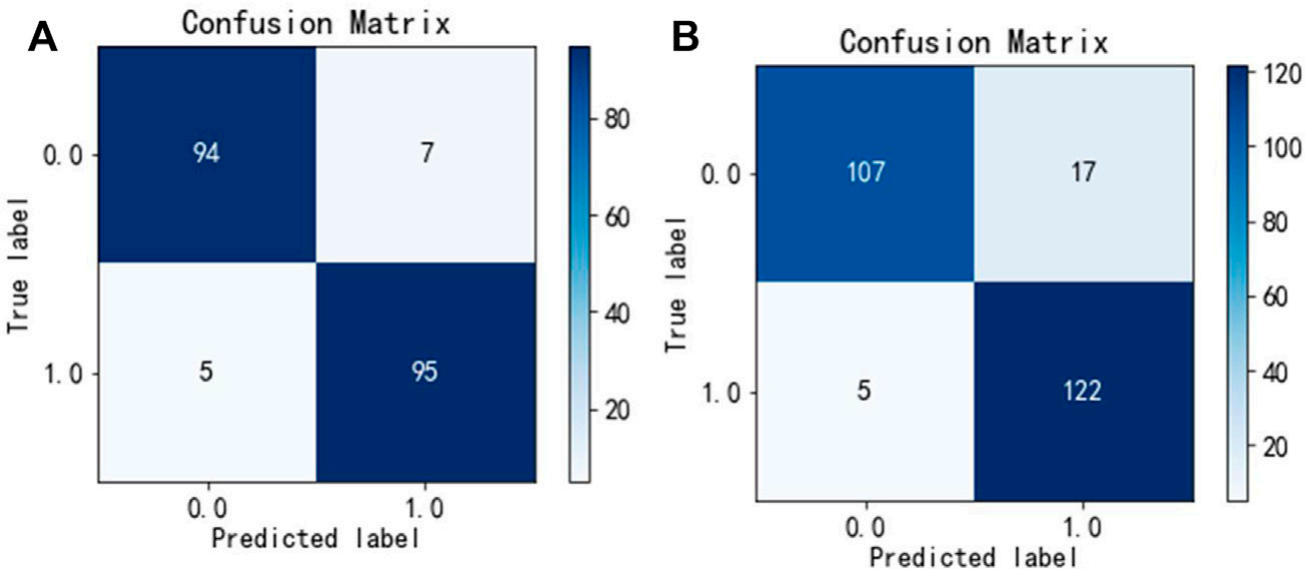
Confusion matrix unde Hybrid_DBP **(A)** validation set, **(B)** test set.

To test the generalization ability of the Hybrid_DBP, we used PDB186 to test Hybrid_DBP. The first step in the process was to extract feature vectors using the monoDiKGap method and Kmer, followed by filtering the extracted feature set with MRMD2.0 to generate the best hybrid features with low redundancy and high relevance. The hybrid features were not only more expressive, but also reduce feature dimensionality. After using CNN for classification, 91.2% of DBPs were identified. Figure 4 showed the confusion matrix for the validation and test sets of the model. According to the result, the model developed in this paper was extremely useful for identifying DBP.

### 3.5 Performance comparison of different classifiers

**FIGURE 5:**
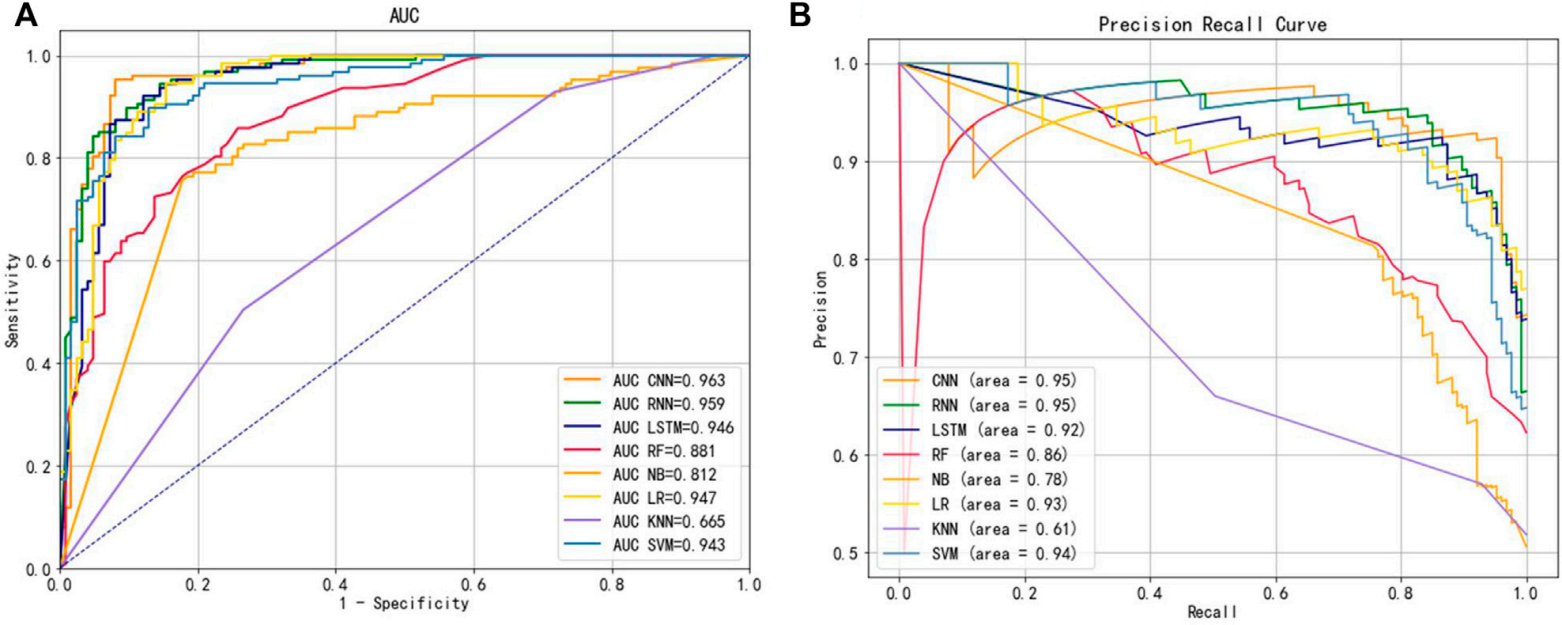
**(A)** ROC curves for different classifiers **(B)** PRC curves for different classifiers.

To explore the extracted features used to select the best classification method, we kept the other conditions of the model constant and explored the model with three classical deep learning methods and five machine learning classification methods while exploring the model. We selected a total of eight widely used classifiers for comparison on the same benchmark dataset, namely CNN, RNN, LSTM, RF, NB, LR, KNN and SVM, based on the Hybrid_DBP approach. Figure 5 illustrated the ROC curve and PRC curves from multiple classification models, in which it could be observed that the ROC curve of CNN should be the furthest from the dotted line, close to the upper left corner, with a value of 0.963. A CNN model with PRC curve value of 0.95, closest to the upper right corner, had the best classification capability. Therefore, CNN was used to construct our final classification model.

### 3.6 Comparison with previous approaches on the independent test set

To compare and analyze the advantages of this model with previous results, we used PDB186 to conduct experiments. Table 2 compared the performance method with 10 previous methods on the independent data set. From the monoTriKGap-CNN method, we found that the ACC was 91.2%, the MCC was 0.828, the SN was 96.1, which were 5.1%, 0.107 and 11% better than the current optimal PsePSSM+PSSM-AB+PSSM-DWT methods, respectively. Compared to most of the existing methods, the CNN-based algorithms performed better at certain confidence levels. The selected method presented in this study is effective and accurate at identifying DBP based on the previous experimental results.

**TABLE 2:** Performance comparison of existing methods

**Method**	**ACC(%)**	**MCC**	**SN (%)**	**SP (%)**
IDNA-Prot|dis	72.0	0.445	79.5	64.5
IDNA-Prot	67.2	0.344	67.7	66.7
DNA-Prot	61.8	0.240	69.9	53.8
DNAbinder	60.8	0.216	57.0	64.5
DNABIND	67.7	0.355	66.7	68.8
DNA-Threader	59.7	0.279	23.7	95.7
DBPPred	76.9	0.538	79.6	74.2
IDNAPro-PseAAC	71.5	0.442	82.8	60.2
Kmer1+ACC	71.0	0.431	82.8	59.1
PsePSSM+PSSM-AB+PSSM-DWT	86.1	0.721	85.1	86.9
Hybrid_DBP	91.2	0.828	96.1	86.1

## 4 Conclusion

The ability to accurately predict DNA-binding proteins could be beneficial for treating diseases, which is more beneficial to developing drugs and treating diseases. This study focuses on the accurate prediction of DBP. The results showed that the best feature set produced by combining monoDiKGap and Kmer *via* MRMD2.0 under convolutional neural networks could predict 94% of DNA binding proteins. Furthermore, with the Hybrid_DBP, 91.2% accuracy was achieved in the independent test set. As a result, the Hybrid_DBP model was a useful method for studying DBP and providing reference values for other research studies.

## Data Availability

The original contributions presented in the study are included in the article/Supplementary Material, further inquiries can be directed to the corresponding author.
